# Toward Smart Biomimetic Apatite-Based Bone Scaffolds with Spatially Controlled Ion Substitutions

**DOI:** 10.3390/nano13030519

**Published:** 2023-01-28

**Authors:** Edoardo Cianflone, Fabien Brouillet, David Grossin, Jérémy Soulié, Claudie Josse, Sanjana Vig, Maria Helena Fernandes, Christophe Tenailleau, Benjamin Duployer, Carole Thouron, Christophe Drouet

**Affiliations:** 1 CIRIMAT, Université de Toulouse, CNRS, INP-ENSIACET, 31030 Toulouse, France; 2 CIRIMAT, Université de Toulouse, CNRS, UT3 Paul Sabatier, 31062 Toulouse, France; 3 Centre de Microcaractérisation Raimond Castaing, Université de Toulouse, UPS, CNRS, INP, INSA, 31400 Toulouse, France; 4 Faculdade de Medicina Dentaria, Universidade do Porto, Rua Dr Manuel Pereira da Silva, 4200-393 Porto, Portugal; 5 LAQV/REQUIMTE, University of Porto, 4160-007 Porto, Portugal

**Keywords:** biomimetic apatite, core-shell particles, controlled ion substitutions, freeze-casting, freeze drying, bone regeneration, antibacterial

## Abstract

Biomimetic apatites exhibit a high reactivity allowing ion substitutions to modulate their in vivo response. We developed a novel approach combining several bioactive ions in a spatially controlled way in view of subsequent releases to address the sequence of events occurring after implantation, including potential microorganisms’ colonization. Innovative micron-sized core-shell particles were designed with an external shell enriched with an antibacterial ion and an internal core substituted with a pro-angiogenic or osteogenic ion. After developing the proof of concept, two ions were particularly considered, Ag^+^ in the outer shell and Cu^2+^ in the inner core. In vitro evaluations confirmed the cytocompatibility through Ag-/Cu-substituting and the antibacterial properties provided by Ag^+^. Then, these multifunctional “smart” particles were embedded in a polymeric matrix by freeze-casting to prepare 3D porous scaffolds for bone engineering. This approach envisions the development of a new generation of scaffolds with tailored sequential properties for optimal bone regeneration.

## 1. Introduction

Bone is a composite calcified tissue composed of an organic collagen matrix mineralized with nanocrystalline non-stoichiometric apatite [1]. The inorganic contents of an average long bone reach about 70 wt.% [2], thus constituting an essential component of bone matter. Bone apatite not only improves the mechanical properties [3], but also plays an active role in homeostasis by interacting with the body fluids [4], which includes the retention and release of bioactive ions as necessitated by the organism [5]. This is made possible by the high (surface) reactivity of bone apatite, which is constituted of sub-stoichiometric nanocrystals exhibiting a non-apatitic hydrated ionic layer containing labile ions [2,6,7]. Previous studies have indeed explored in detail the physicochemical and thermodynamic characteristics of either natural or synthetic (biomimetic) apatites [1,2,3,4,5,8,9]. Provided that bio-inspired precipitation methods are used, biomimetic apatites thus offer a particularly relevant platform for the setup of bone substitutes.

Taking into account the potentialities of the apatite structure to accommodate numerous types of ion substituents, it then becomes possible to prepare functionalized apatites with added properties by adequately selecting the substituting ions. In this view, one appealing strategy can consist of conferring the antimicrobial/antibacterial properties in link with the high infection risks in bone surgery, such as in orthopedics or dentistry [10]. These risks are all the more crucial to consider due to the massive bacterial resistance effects observed toward the main antibiotic agents [11]. Such antimicrobial resistance (AMR) has become a major threat in public health: in 2019, for example, it led to *ca*. 33,000 deaths within the European Economic Area (EEA) and generated 1.1 billion euros of related healthcare costs [12,13]. By 2050, unless strong actions are developed, it is anticipated that AMR could globally cause more deaths than cancers [14], with an economic payload similar to the 2008 financial crisis estimated by the World Bank [15].

In order to provide antimicrobial/antibacterial functionalities to apatite compounds, several key elements can be envisioned. Metallic silver (Ag^0^) is sometimes used in composite systems with hydroxyapatite [16,17]. On the other hand, ionic species such as (Ag^+^) [18,19,20] and copper (Cu^2+^) [17,20,21,22] have also shown particular promise in this field. Conferring antimicrobial activity through ionic species as an alternative strategy to antibiotics indeed appears to be a promising route to fight against AMR.

Beyond antimicrobial/antibacterial properties, bone scaffolds could advantageously benefit from other functionalities in view of favoring bone healing, which also covers the stimulation of osteogenesis (the activation of osteoblast cells and the promotion of osteointegration), as well as neo-angiogenesis (the activation of endothelial cells and the formation of new blood capillaries). Cu^2+^ ions have proven to exhibit an interesting angiogenic behavior since at least 1980 [23,24] and the interest in them is extremely vivid [25,26,27,28]. The use of pro-angiogenic/osteogenic ions such as Cu^2+^ appears as a relevant strategy compared to the use of growth factors, which is debated in the scientific community, as it is linked with reported side effects such as their potential carcinogenicity [29,30]. Other ions were also found to be bioactive and appear promising for the setup of bone substitutes capable of stimulating bone neoformation. Among these, Zn^2+^ ions proved particularly well-suited for stimulating bone regeneration [31,32,33,34] thanks to anti-inflammatory [35] and osteogenic intrinsic features.

Although various ions can thus bring promise in the field of bone regeneration and have been the object of various studies, only a limited amount of data are available on the combination of several bioactive ions in apatite-based compounds [36]. Indeed, in the literature so far, mainly two types of multi-substituted apatites have been addressed: those involving multiple substituting cations in Ca^2+^ sites [37,38,39,40,41], and those presenting a combined anion/cation substitution in both Ca^2+^ and PO_4_^3−^/OH^−^ sites [20,42,43,44,45]. Also, within the accessible literature on this topic, the general approach consists in direct co-substituting, with no cumulated control of ion release in time and space. While multilayer coatings aiming at the delivery of diverse biological molecules such as growth factors have been described [46], no similar approach is available to date for the controlled time/space delivery of mineral ions.

In this contribution, we aimed to develop an innovative strategy based on the preparation and use of biomimetic apatites associated with several bioactive cations in a controlled spatial way in view of subsequent release features. Considering the sequence of events expected to occur after implantation [47,48,49], the goal is to first deliver an antibacterial ion to eradicate pathogenic microorganisms, followed by the subsequent release of a pro-angiogenic and/or osteogenic ion to stimulate bone healing. Such release events could further be facilitated by the often-encountered acidic pH found in inflammatory conditions [50,51,52]. We report herein the physicochemical background of this proof of concept by designing different core-shell apatite particles generated via a 3-fluid nozzle spray drying approach. We incorporated such engineered particles into 3D biopolymer scaffolds exhibiting an oriented porosity to favor bone cell colonization and bone repair, prepared by freeze-casting. The systems exhibiting silver ions (antibacterial) on the outer shell and copper ions (pro-angiogenic/osteogenic) in the inner core appear particularly suited for our aimed concept. Since Ag^+^ ions are not naturally present in vivo (contrary to Cu^2+^ or Zn^2+^), preliminary in vitro tests were run to confirm that Ag-substituted biomimetic apatite remained cytocompatible with osteoblasts, while proving antibacterial toward both Gram negative (*E. coli*) and Gram positive (*S. aureus*) strains.

## 2. Materials and Methods

### 2.1. Synthesis of Biomimetic Apatites

The biomimetic apatite compounds used in this work were prepared in close-to-physiological conditions, at room temperature and pH 7.2. This pH value was obtained naturally via the use of an excess of phosphate reagent, thus preventing the use of an external buffering agent such as TRIS. A 0.3 M calcium solution (750 mL) was prepared by dissolving calcium nitrate tetrahydrate Ca(NO_3_)_2_^.^ 4H_2_O (Merck—Emsure^®^, Darmstadt, Germany) in deionized water. A 0.6 M phosphate solution (1500 mL) was prepared by dissolving di-ammonium hydrogen phosphate (NH_4_)_2_HPO_4_ (Carlo Erba, Val de Reuil, France) unless otherwise specified. After pouring the less concentrated solution into the more concentrated one, the mixture was stirred (700 rpm, 5 min) to ensure an identical maturation state for all the crystals formed and the precipitation medium was left to age for 1 day. The apatite precipitate was then separated by filtration in a Büchner funnel and washed with deionized water. In view of the 3-fluid nozzle spray drying approach, the precipitate was mixed with water with a precipitate/water mass ratio of 1:1.15 to prepare the apatite gels with a mean concentration in a solid phase of ~45 mg/mL. When needed in the spray drying process, additional dilution of such gels could be performed to optimize flowability in the setup. For characterization purposes, the powdered samples were also prepared by direct freeze-drying (−80 °C, 10 mbar) of the precipitate after the washing step, and the powders were then stored in a freezer at −18 °C prior to further use.

Incorporation of silver, copper or zinc ions in the apatite samples was reached by substituting the desired molar content of Ca^2+^ in the protocol above by the corresponding amount of the target ion. The reactants used were as follows: silver nitrate (AgNO_3_, Alfa Aesar, Kandel, Germany), copper nitrate (Cu(NO_3_)_2_^.^ 3H_2_O Acros Organics, Geel, Belgium) and zinc nitrate (Zn(NO_3_)_2_^.^ 6H_2_O Alfa Aesar). In the case of copper and zinc substituting, the (NH_4_)_2_HPO_4_ reactant was replaced by its sodium counterpart Na_2_HPO_4_ (Merck—Emsure^®^) to avoid detrimental secondary phases, especially at a high substituting rate (unpublished data).

### 2.2. Three-Fluid Nozzle Spray Drying

**Figure 1:**
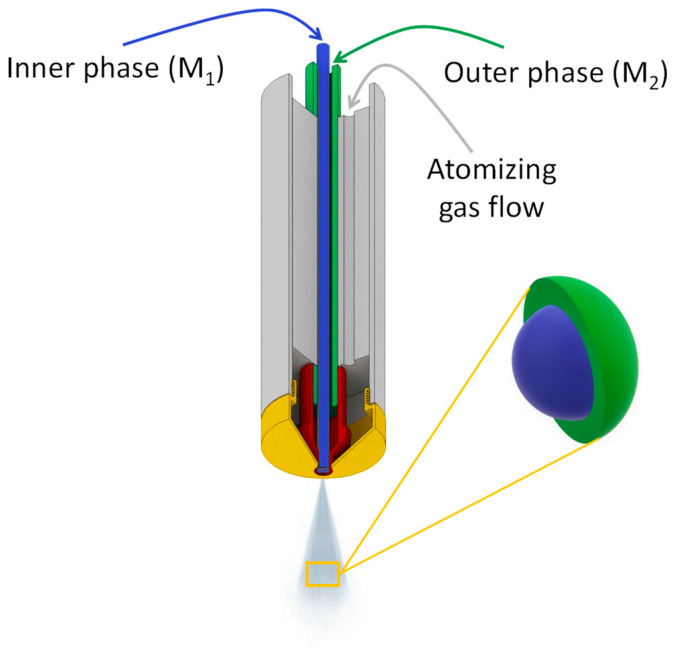
Three-fluid nozzle spray drying schematic.

In view of the production of core-shell apatite particles, a methodology was set up using a Büchi Spray Dryer B-290 apparatus equipped with a 3-fluid nozzle [53] for the independent feeding of two diversely substituted apatite gels (Figure 1). Air (fluid 1) was used as an atomizing gas at a flowrate of 667 NL/h. The aspiration rate of the apparatus was set to 90%. The drying temperature was 210 °C. The global flowrate for feeding the two other fluids (fluids 2 and 3) was 4.3 mL/min corresponding to 3.3 mL/min in the outer phase using the built-in pump and 1 mL/min in the inner phase introduced by an additional peristaltic pump. The apatite gel used in the outer phase was substituted with an antibacterial ion (Ag^+^ or Cu^2+^), whose release is expected first, while the inner phase consisted in an apatite gel substituted with an osteogenic and/or pro-angiogenic ion (Cu^2+^ or Zn^2+^) expected to play a subsequent role in the bone repair process. In this work, the notation [(M_1_^n+^)M_2_^m+^] will be used, where the first ion mentioned (here M_1_^n+^) is the one located in the inner core, while the second ion cited (M_2_^m+^) is the one contained in the outer shell.

### 2.3. Preparation of Freeze-Cast Porous Scaffolds

Porous scaffolds exhibiting an oriented porosity were obtained by freeze-casting using cylindrical molds (2 cm diameter, 5 cm height) obturated by copper caps for optimal heat transfer. The mold was isolated from its surroundings thanks to an insulating polymeric foam envelope. The evolution of the freezing front from bottom to top aiming at providing the desired oriented porosity was made possible by connecting the lower part of the mold to a copper cold finger in a homemade freeze-casting device set to −20 °C. The molds were partially filled with a suspension of the engineered apatite microparticles in a polymeric solution (sodium alginate, Sigma Aldrich, Saint-Quentin-Fallavier, France), dissolved in deionized water to a mass proportion of 3.5 wt.%). The mass ratio between the apatite core-shell particles and alginate was set to 40:60. After adding the particles in the alginate solution, the suspension was homogenized by Ultra Turrax^®^ (45 s at 21,000 rpm). A total of 4.6 mL of suspension were then introduced in the mold and left to freeze-cast for 3 h. The molds were then opened, and the frozen suspension was lyophilized for 24 h (Christ Alpha 2-4 LD2, Osterode am Harz, Germany) at −80 °C, 0.05 mbar.

### 2.4. Physicochemical Characterization

The crystalline structure of the apatite samples was verified by X-ray diffraction (XRD) using a Bruker D8 diffractometer (copper anticathode Kα, λ_Cu_ = 1.5418 Å) in the 2θ range 20–80° with a step of 0.02° for an acquisition time of 1 sec. Data processing was carried out on the DIFFRAC.EVA^®^ software V3.1 (Bruker, Karlsruhe, Germany) and Origin Pro^®^ version 2021b (Northampton, MA, USA).

The complementary structural information was obtained by Fourier transform infrared (FTIR) spectroscopy, using the KBr pellet transmission methodology, on a Nicolet IS50 spectrometer in the 400–4000 cm^−1^ wavenumber range, with 64 scans of acquisition at a resolution of 4 cm^−1^.

In order to determine the substituting ratios of the apatite samples, elemental titrations of the cations (Ca^2+^, Ag^+^, Cu^2+^, Zn^2+^, Na^+^) were carried out by atomic absorption spectrometry (AAS) using a Thermo Scientific ICE 3000 (Waltham, MA, USA). Prior to the analysis, each sample was dissolved in acidic conditions with 2% nitric acid from a stock NHO_3_ solution at 69%. Except for the silver-containing samples, 0.5% La(NO_3_)_3_ and 0.5% CsCl were used as matrix modifiers for analysis optimization. For the Ag-substituted samples, 1% La(NO_3_)_3_ was used and in the absence of CsCl (since the presence of chlorine ions would lead to AgCl secondary precipitation). The analysis wavelengths for Ca, Ag, Cu, Zn and Na were 422.7, 328.1, 324.8, 213.9 and 589.0 nm, respectively. For each element, a calibration line was established in identical experimental conditions, starting from stock standard solutions (SCP SCIENCE, Baie-d’Urfé, QC, Canada), with a minimal correlation factor of 0.995. The data were processed with the SOLAAR^®^ software V11.11 (Waltham, MA, USA).

When relevant, particle size distribution analyses in the dry state (AeroS^®^ measuring cell) were carried out on the apatite powders by laser diffraction using a Malvern Mastersizer 3000 apparatus (wavelength λ = 633 nm). The distributions are given in terms of volumetric D_50_ representing the median size distribution. Helium pycnometry experiments were also carried out to evaluate the apparent density of some of the selected compounds. To this aim, a Micromeritic AccuPyc II 1340 apparatus was used, and data processing was performed with the AccuPyc II 1340 V1.09 software (Norcross, GA, USA).

The morphological observations of both the apatite particles and the freeze-cast scaffolds were performed by scanning electron microscopy on a FEI (Hillsboro, OR, USA) Quanta 450 microscope operated at 12.5 kV, in secondary electron mode. To limit alteration of the samples and the accumulation of electrons due to their insulating character, the analyses were carried out in a low vacuum mode by using a residual water vapor pressure of 90 Pa.

The samples were investigated using a FIB FEG-SEM coupled with energy-dispersive spectroscopy (EDS). The apparatus used was a FEI Helios NanoLab 600i coupled to an Aztec Oxford EDS system (SDD detector, WD 4 mm). Before the SEM observation, the samples were coated with an amorphous carbon layer applied by sputtering (Leica ACE 600, Wetzlar, Germany), with a film thickness of around 5–10 nm to ensure the sample conductivity. In situ cross sections were produced by focused ion beam (FIB) milling using Ga^+^ ions. The sample was tilted at 52° and a platinum (Pt) layer with a width of about 1 μm was laid down for protection on the selected surface. An ionic ablation of Ga^+^ ions was then applied on the surface, producing cross sections from 20 to 40 μm in length and 10 to 20 μm in depth. FEG-SEM observation and semi-quantitative EDS analysis of the cross sections were then carried out using an acceleration voltage of 20 kV.

The 3D porous structure of the freeze-cast scaffolds was followed by X-ray computed microtomography (µCT). The images were acquired with a Phoenix/GE Nanotom 180 instrument from the French Research Federation FERMAT (FR3089) equipped with a tungsten target operated at 60 kV/300 µA and an HCD-5184-50 detector (2300 × 2300 pixels). The samples were positioned at 62 mm from the X-ray target. A 300-mm distance was set between the source and the detector, with a counting time of 1 s per picture and an average of seven pictures per step. Data processing was performed with the Datos X^®^ software (GE Sensing & Inspection Technologies GmbH, Phoenix|X-ray, Wunstorf, Germany ), allowing 3D reconstructions of the scaffolds. The images were treated with the Vg Studio Max^®^ software 2.1 (Volume Graphics GmbH, Heidelberg, Germany); the maximum voxel size was of 10.3 μm.

### 2.5. Biological Evaluations

#### 2.5.1. Osteoblastic Cytocompatibility of Cu- and Ag-Substituted Apatite Powders

Cu- and Ag-substituted apatite particles (with a 0.2% and 0.5% substitution ratio, respectively) were subjected to 30 min of UV sterilization. The particles suspensions were prepared in basal alpha-MEM medium at five different concentrations (1 µg/mL, 10 µg/mL, 100 µg/mL, 250 µg/mL and 500 µg/mL) followed by sonication for particle dispersion. Osteoblastic MG63 cells (ATCC^®^ CRL-1427) were seeded at a concentration of 10^4^ cells/cm^2^ in alpha-MEM medium supplemented with 10% (*v*/*v*) fetal bovine serum (FBS), 100 U/mL penicillin, 100 µg/mL streptomycin and 0.25 µg/mL amphotericin B (all from Gibco, Waltham, MA, USA). The particle suspension was constantly stirred during seeding to maintain a homogeneous concentration. After 24 h, which is necessary for cell adhesion, the culture medium was replaced by a different one containing the sterilized apatite particles (1 to 500 µg/mL). The apatite-exposed cells were cultured for 1, 3 and 7 days, at 37 °C, in a humidified atmosphere of 5% CO_2_/air. The cultures performed in the absence of apatite powders were used as controls. The cell behavior was evaluated for cell viability (MTT assay) and accessed for morphology by fluorescence microscopy after being stained for the nucleus, F-actin cytoskeleton and mitochondria tracking.

#### 2.5.2. MTT Assay

In this assay, mitochondrial dehydrogenase in viable cells is able to reduce the MTT reactant [3-(4,5-dimethylthiazol-2-yl)-2,5-diphenyltetrazolium] to a dark blue formazan product. The cultures were incubated with MTT (0.5 mg/mL, Sigma-Aldrich) for 3 h at 37 °C. Then, the culture medium was removed, and the formazan salts were dissolved in dimethyl sulphoxide (DMSO, Panreac, Castellar del Vallès, Spain), and the absorbance was determined at λ = 550 nm on a microplate reader (Synergy HT, Biotek, Winooski, VT, USA). A parallel experiment was run with the particles being incubated in culture medium (without cell seeding), using the same protocol as for the cell cultures, to offset any MTT reduction by the particles themselves.

#### 2.5.3. Cell Morphology and Mitochondria Tracking

At the end of each time-point, the cells were incubated for 30 min with MitoSpy™ Red FM at 37 °C and 5% CO_2_. The cells were then fixed in paraformaldehyde 3.7% following their permeabilization with Triton-X 0.1% and incubation with 1 wt.% bovine serum albumin (BSA, Sigma-Aldrich, 30 min) to avoid non-specific interactions, at room temperature. Finally, the cells were stained with phalloidin-conjugated Alexa Fluor^®^ 488 (1:100, Molecular Probes (Eugene, OR, USA), 30 min) and Hoechst 33342 (Enzo Life Sciences (Villeurbanne, France), 10 min) for F-actin and nucleus staining, respectively. The images of the stained cells were acquired using a Celena S digital imaging system (Logos Biosystems, Anyang, Republic of Korea).

#### 2.5.4. Osteoblastic Cytocompatibility of Ag-Substituted Apatite Pellets

The apatite (hap) and 0.5% silver-substituted apatite pellets (hapAg 0.5%) were UV sterilized (30 min) and pre-incubated in complete culture medium (composition described above) for 1 h. Then, they were seeded with MG63 osteoblastic cells (2 × 10^4^ cells/cm^2^) and cultured for 4 and 7 days, as above. The seeded pellets were characterized for cell adhesion and morphology (day 4, SEM), metabolic activity (days 4 and 7, Resazurin assay) and alkaline phosphatase (ALP) activity (day 7).

#### 2.5.5. Resazurin-Based Assay

Briefly, 10% (*v*/*v*) resazurin solution (Sigma-Aldrich, St. Louis, MO, USA), 0.1 mg/mL, was added to the seeded pellets. After 3 h of incubation at 37 °C, the metabolic activity was evaluated by measuring the fluorescence of the medium at λex: 530 nm and λ_em_ = 590 nm on a microplate reader (Synergy HT, Biotek). The results were expressed as relative fluorescence units (RFUs).

#### 2.5.6. ALP Activity

The cell lysates (0.1% *v*/*v* Triton X-100, 30 min) of the seeded pellets (7-day cultures) were evaluated for ALP activity by the hydrolysis of p-nitrophenyl phosphate (pH~10.3, 1 h, 37 °C), and assessment of the formed p-nitrophenol (λ = 400 nm; ELISA reader, Synergy HT, Biotek). ALP activity was normalized to total protein content (DCTM Protein Assay, BioRad, Hercules, CA, USA), and expressed as nmol/min.µg_protein_^−1^.

#### 2.5.7. SEM

The seeded samples were fixed (glutaraldehyde 1.5% in sodium cacodylate buffer 0.14 M, Sigma-Aldrich, for 15 min), dehydrated in graded alcohol, critical-point dried and sputter coated with a palladium/gold alloy prior to imaging (FEI Quanta 400 FEG/ESEM).

#### 2.5.8. Antibacterial Activity of Ag-Substituted Apatite Pellets

The antibacterial activity of the sterilized apatite pellets (hap and hapAg 0.5%) was evaluated against the Gram-positive *Staphylococcus aureus* ATCC 25923 and the Gram-negative *Escherichia coli* ATCC 25922. The bacterial suspensions, *ca*. 1.5 × 10^8^ cells/mL in TSB (Liofilchem, Roseto degli Abruzzi, Italy), were prepared by optical density, poured over the biomaterial pellets, and cultured for 24 h in a shaker incubator at 37 °C and 100 rpm. The bacteria cultured in the absence of the pellets were used as a control. The antibacterial activity was characterized for the metabolic activity (Resazurin assay, as described above) of planktonic and sessile bacteria, respectively, bacteria in suspension and those adhered to the apatite pellets. Then, the colonized pellets were sonicated to displace the adhered bacteria and the resultant bacterial suspensions were plated in tryptic soy agar (TSA, Liofilchem) plates and incubated at 37 °C for 24 h to further evaluate the number of cultivable bacteria (colony-forming units, CFUs).

#### 2.5.9. Statistics

The results are presented as mean ± standard deviation of three independent experiments, with three replicas each. Statistical analyses were performed by one-way analysis of variance (ANOVA), in combination with Tuckey’s post hoc test. Values of *p* ≤ 0.05 were considered significant.

## 3. Results and Discussion

### 3.1. Substituting Bioactive Ions in Biomimetic Apatites

In the first stage, the substituting of 1-day matured biomimetic apatite with a bioactive ion type selected among Zn^2+^, Cu^2+^ or Ag^+^ was carried out so as to obtain single-substituted apatite particles, characterize their main physicochemical features, and unveil potential substituting effects compared to the unsubstituted reference sample. Several substituting contents were investigated, as listed in the second column of Table 1 (theoretical substituting ratios). While the low substituting rates are intended for biological applications, the selection of some higher substitution ratios was made to facilitate characterization of the core-shell particles to be prepared, and thus validate our spray drying approach.

**Figure 2:**
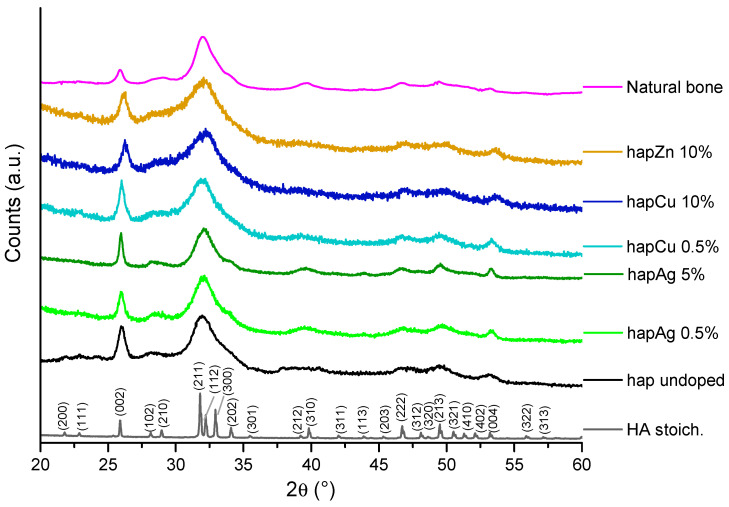
XRD patterns of the selected precipitated biomimetic apatite samples, as well as reference stoichiometric HA, undoped apatite and natural bone (rat, 9 months old, internal CIRIMAT collection). The (hkl) indexation refers to the HA phase (PDF file # 00-009-0432).

The XRD patterns of all the precipitated samples show their apatitic structure by the good general accordance with the hydroxyapatite “HA” reference (hexagonal, P6_3_/m space group, PDF file # 00-009-0432). Figure 2 reports a selection of relevant XRD patterns. A moderate degree of crystallinity may, however, be noticed for the precipitated samples, in agreement with previous data [8], as is also found for natural bone. As already reported, this lower crystallinity state may be related to both the nanocrystalline character of the apatite crystals and to the microconstraints in the apatite unit cell. Also, the presence of substituting ions may generate additional distortions in the unit cell. Application of Scherrer’s equation to the illustrative example of sample hapAg 0.5% leads to a mean crystallite length along the *c*-axis of *ca*. 18 nm and a mean width/depth of *ca*. 6.5 nm, confirming its nanocrystalline nature. It may be noted that no secondary crystalline phases were detected in the present study, even for the highest substitution ratios. However, we may highlight a peak broadening effect upon increasing the substitution rate, which may be related to the distortion phenomena mentioned above. It also demonstrates that the substituting ion was efficiently incorporated into the apatite structure [7,54].

**Figure 3:**
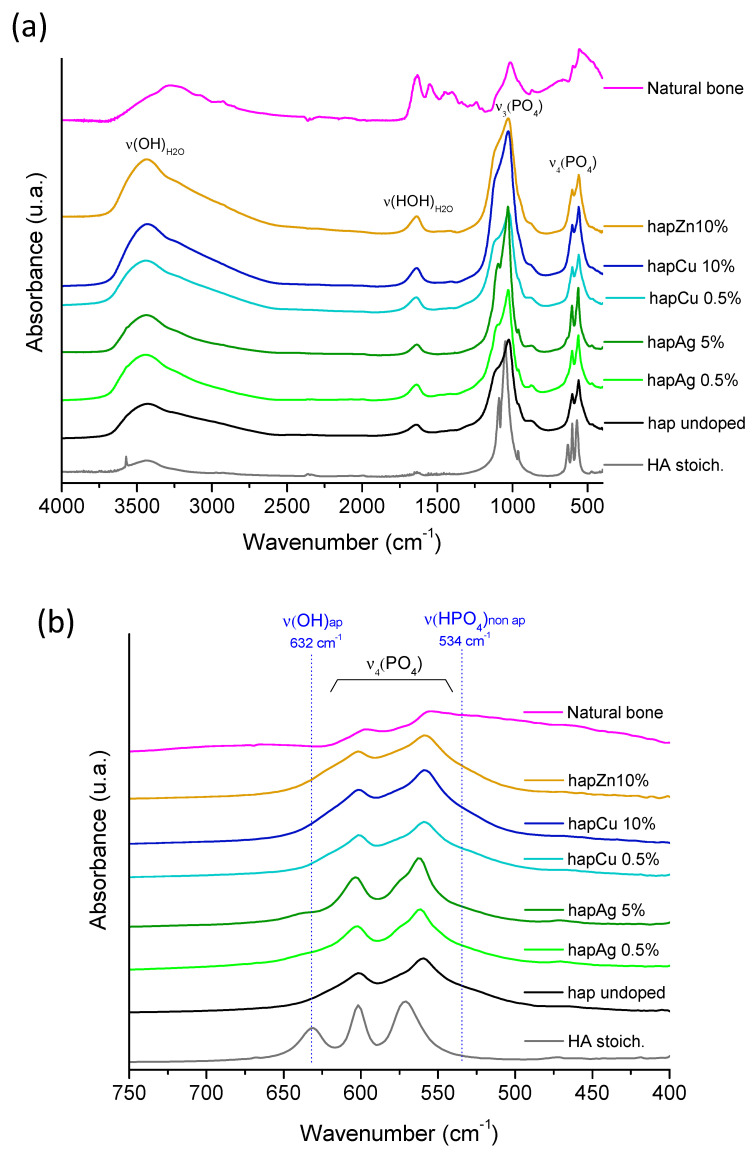
(**a**) FTIR spectra of the selected precipitated biomimetic apatite samples, as well as reference stoichiometric HA, undoped apatite and natural bone (rat, 9 months old, internal CIRIMAT collection), (**b**) zoom in the spectral range 400–800 cm^−1^.

Complementary characterization was provided by the FTIR analyses, which provide further information on local chemical environments (Figure 3). All the spectra were characteristic of bone-like apatites without any additional unexpected absorption bands. Of particular relevance is the noticeable presence of an absorption shoulder at 534 cm^−1^, assignable to the non-apatitic surface HPO_4_^2−^ ions [7], as well as the low intensity of the OH^−^ libration band (i.e., a vibration mode involving the rotation of the OH ion around an equilibrium position) at 632 cm^−1^. Both of these observations point to the sub-stoichiometric character of these synthetic apatite phases, as in bone mineral, thus confirming their biomimetic character. It should be noted that a lower IR band resolution was observed for higher substitution rates—as visible, for example, in the ν_3_PO_4_ domain—which may be related to the lower crystallinity of these samples, as evidenced by XRD.

Titration of the cations was carried out by AAS, with the view to determine the effective substitution rates, and the related data can be found in the third column of Table 1 (experimental substituting ratios). A good agreement, within 5%, is found with the nominal values, except for high initial Ag contents (e.g., 5 mol.%) with up to 50% departure from the theoretical values, which may be related to the monovalent character of Ag^+^ and/or to a significantly different thermodynamic behavior compared to Ca^2+^. As a reminder, the thermodynamic properties of both the aqueous species and the substituted apatite phases likely depend on the nature of the substituting ion, as shown previously [55].

The SEM observations were performed to examine the morphology of such single-ion substituted apatite compounds. The obtained micrographs (Supplementary Materials, Figure S1) show polycrystalline particles with no specific shape, and a large distribution in size in all cases. For indicative purposes, laser diffraction analyses allowed us to determine the D_50_ median size to *ca*. 1.1 µm for the unsubstituted apatite and *ca*. 1 µm for high substituting rates (e.g., for Cu 10% initial substituting).

### 3.2. Core-Shell Multi-Substituted Particles via 3-Fluid Nozzle Spray Drying

**Figure 4:**
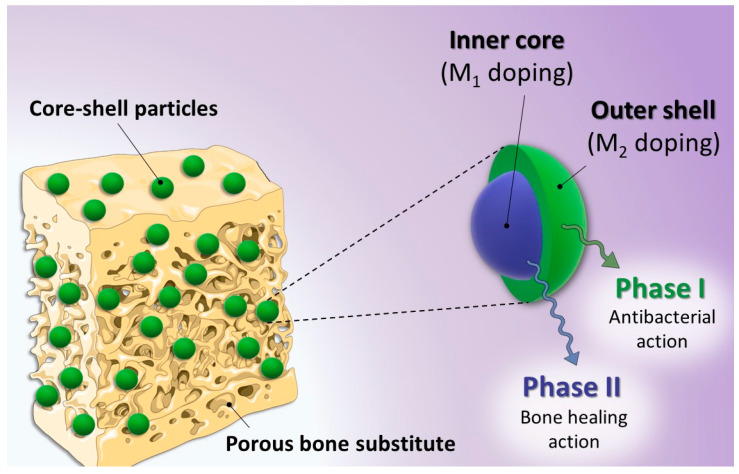
Overview of the proof of concept based on core-shell multi-substituted apatite with the controlled distribution of different bioactive ions.

The findings above suggest the possibility to prepare single-ion substituted biomimetic apatite samples substituted with bioactive Ag^+^, Cu^2+^ or Zn^2+^ ions, with a large range of substituting ratios and retaining the main physicochemical features compared to their non-substituted counterpart or natural bone apatite. These conclusions established the background knowledge in view of our proof of concept aiming to prepare dually-substituted apatite particles with a spatial control on the location of the substituting ions via the formation of core-shell structures. In this view, the 3-fluid nozzle spray drying technology appeared particularly well-suited to the obtainment of such layered particles. In this approach, an apatite gel substituted with an osteogenic and/or pro-angiogenic ion (typically Cu^2+^ or Zn^2+^) was used in the inner core, while a second apatite gel substituted with an antibacterial ion (e.g., Ag^+^ or Cu^2+^) constituted the outer shell (Figure 4).

The first core-shell system that we investigated was [(Zn^2+^)Cu^2+^], in which Zn^2+^ was located in the inner core, while Cu^2+^ was contained in the outer shell. In order to facilitate the characterization of the final product, a rather high substituting content of 10% (i.e., mole ratio relative to Ca^2+^ in the starting precipitation medium) was used for both gels.

As expected, the particles obtained after spray drying still exhibited the characteristic structural features of biomimetic apatites, as confirmed by XRD and FTIR analyses (see Supplementary Materials, Figure S2), with the absence of detectable secondary phases. The data point to rather similar apatitic features was compared to the corresponding single-substituted apatites, except for a lower degree of crystallinity—as seen by XRD peak broadening—which may be related to the very high drying kinetics undergone in the spray drying process.

**Figure 5:**
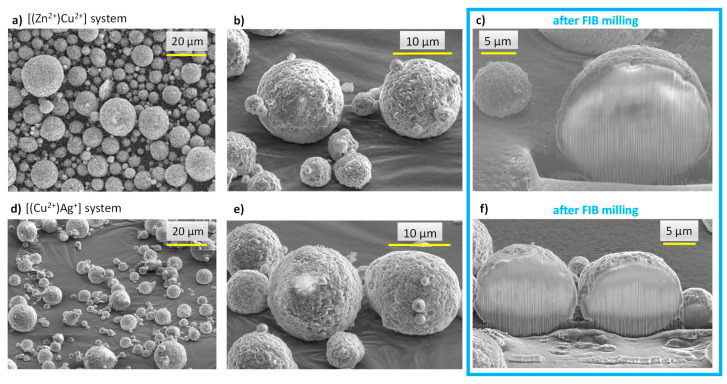
SEM micrographs of spray-dried core-shell particles: images (**a**–**c**): [(Zn^2+^)Cu^2+^] system, images (**d**–**f**): [(Cu^2+^)Ag^+^] system. Images (**c**,**f**) refer to observations made after FIB milling.

Morphological analyses were carried out by SEM on the spray-dried [(Zn^2+^)Cu^2+^] particles, as depicted in Figure 5a,b. A clear spherical shape can be noticed for all the particles obtained by this process, which may be linked to the fast drying of the globular droplets generated at the edge of the 3-fluid nozzle. Processing of the micrographs with the ImageJ^®^ software (free software, NIH, USA) allowed for the evaluation, for this sample, of the particle size spanning 1.5–15 µm with a Gaussian statistical distribution (considering 250–300 particles) leading to a mean diameter of 6.0 ± 3.3 µm (Figure S3).

**Figure 6:**
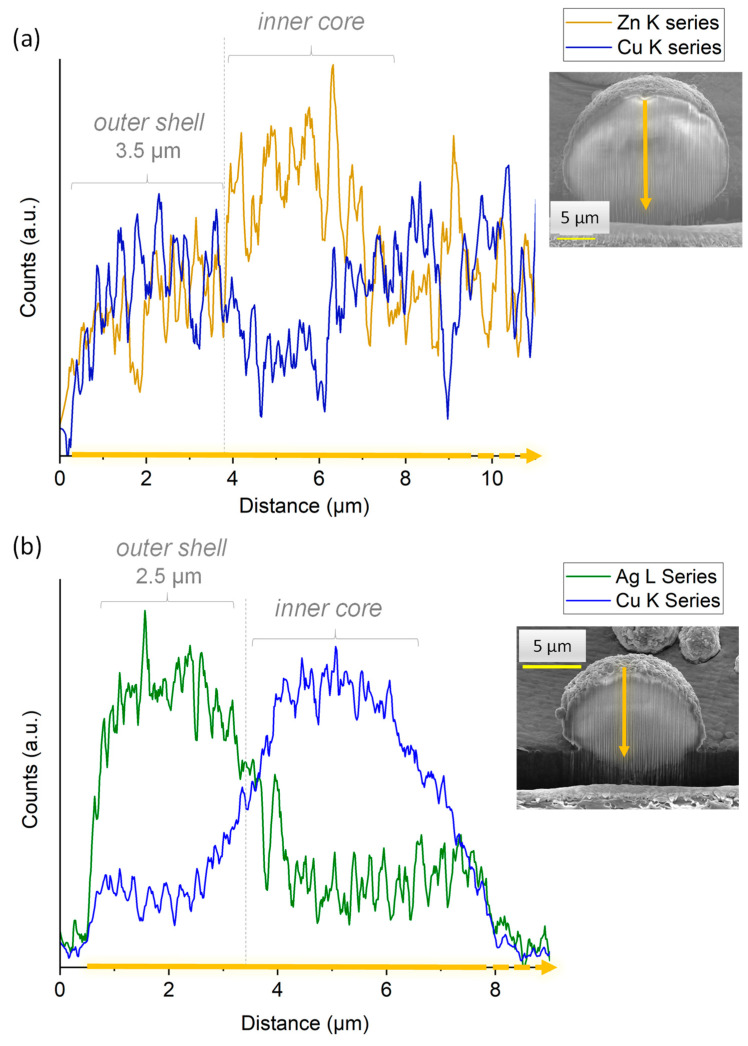
EDS analyses of (**a**) [(Zn^2+^)Cu^2+^] and (**b**) [(Cu^2+^)Ag^+^] particles cross-sections generated in situ by FIB milling with Ga^+^ ions.

In order to examine the internal structure of the particles, in situ cross-sections were produced by FIB milling using Ga^+^ ions and observed by FEG-SEM (Figure 5c). As can be seen, after FIB milling, the particles exhibited a rather dense structure with only a very limited presence of pores. With the aim to examine the location of the substituting ions within the particles, EDS analyses were carried out. It should, however, be noted that such measurements have a non-negligible depth of penetration that can be estimated in our conditions—using Castaing’s formula—to *ca*. 2.5 µm (Figure S4), thus leading to semi-quantitative elemental analyses. In order to limit insofar as possible the bias related to this penetration depth, the analyses were more specially run on 10+ µm particles. Performing EDS analyses along such particles’ cross sections (Figure 6a) allowed us to evidence a dual distribution of the substituting ions, with an external shell enriched with Cu^2+^ ions and an inner core rich in Zn^2+^. These observations are therefore in perfect agreement with the concept pursued in this work (see Figure 4), aiming to produce core-shell particles from two distinct substituted-apatite gels via a concentric nozzle spray drying approach.

In order to further illustrate the feasibility of our core-shell concept, we extended the study to a second system, namely [(Cu^2+^)Ag^+^], thus with the view to expose the highly antimicrobial Ag^+^ ions on the external shell of the particles, which provides a massive antibacterial effect in the first hours after implantation, while Cu^2+^ is known for its proangiogenic/osteogenic properties. As in the previous experiment, FEG-SEM and FIB milling were used to explore the structure and morphology of the particles. As shown in Figure 5d–f, the expected core-shell structuration of the spherical particles was again obtained in these conditions. EDS cross-sectional analyses (Figure 6b) once more evidenced a dual distribution of the substituting ions, with an external layer rich in Ag^+^ ions and an internal composition substituted with Cu^2+^.

Despite the limitations in EDS accuracy in terms of the depth of analysis mentioned above, a first approximation of the mean thickness of the outer shell can be assessed. It may be noted that exploitation of the EDS data becomes less accurate for analyses closer to the sample holder, as it generates numerous additional signals limiting quantitative assessments. For this reason, the shell thickness can be more accurately assessed from the analysis of the opposite part of the particles located farther from the sample holder. For example, from Figure 6a,b, the approximate thickness of the outer shell (after correction due to the tilt of 52°) in both [(Zn^2+^)Cu^2+^] and [(Cu^2+^)Ag^+^] particles of ~15 µm cross-section can be estimated to ~3.5 µm and ~2.5 µm, respectively.

The thickness of the outer layer of a given particle may also be theoretically calculated by taking into account some fundamental characteristics (composition, unit cell parameters and related phase density) of the 1-day matured biomimetic apatite that was used as a basis for the two gels formulations. Indeed, the volume ratio between the outer shell and the inner core for any given particle can be related to the mass ratio of the two corresponding phases by the relationship:

(1) $$\frac{V_{outer}}{V_{inner}}=\frac{m_{outer}}{m_{inner}}\times \frac{d_{inner}}{d_{outer}}$$

where *d* refers to the density of the dried gels, whether constituting the outer or the inner component. Considering a spherical particle shape of radius R (see notations on Figure 4), as evidenced by the SEM observations, and their substructure consisting of an internal spherical core (of radius X) and a concentric outer shell (of radius R-X), the volume ratio from Equation (1) can be rewritten as:

(2) $$\frac{V_{outer}}{V_{inner}}=\frac{\frac{4}{3}\;\pi R^{3}-\frac{4}{3}\;\pi X^{3}\;}{\frac{4}{3}\;\pi X^{3}}=\frac{R^{3}-X^{3}\;}{X^{3}}$$

By combining Equations (1) and (2), the value of X can be determined from the ratios between the masses and the densities. Considering the [(Cu^2+^)Ag^+^] particles as an illustrative example, the densities were accessed by direct measurements via He pycnometry by measuring the volume of a given mass of the Cu- or Ag-monosubstituted samples (i.e., obtained with the reference spray-drying experiments run with identical substituted compositions for the shell and the core). The mass ratio can be determined from the AAS compositional analyses (i.e., from experimental the Ag/Ca and Cu/Ca ratios for single-substituted particles and the (Ag+Cu)/Ca data for the dually-substituted particles). In our conditions, analysis of the AAS data (Table 1) and cross validation with previous compositional data on 1-day biomimetic apatite obtained in similar experimental conditions [56] lead to a mean particle composition close to:

0.39 (Na_0.17_Ca_7.48_Cu_0.73_(PO_4_)_4.53_(HPO_4_)_1.47_(OH)_0.05_) + 0.61 (Ca_8.41_Ag_0.22_(PO_4_)_4.77_(HPO_4_)_1.23_(OH)_0.28_) (3)

This evaluation suggests a molar ratio of 0.39:0.61 between the Cu-substituted inner core and the peripheral Ag-substituted outer shell, respectively, which corresponds to a mass ratio of 38:62 wt.%. Therefore, considering as an illustrative example the [(Cu^2+^)Ag^+^] particle cross-section of 15 µm (R = 7.5 µm), application of Equation (2) leads to X ~ 5.5 µm, thus leading to a shell thickness of ~2 µm. This value may be compared to the ~2.5 µm estimated above from the EDS analysis after FIB milling. Taking into account the limitations on EDS titration due to the potential influence of underlying ions, and the uncertainty on the chemical composition calculated in Equation (3), these findings may be considered in a good agreement.

These findings thus demonstrate that this 3-fluid nozzle approach is well-suited to prepare micron-sized apatitic particles with a controlled dual distribution of two substituting bioactive ions, with an external shell and an internal core, that are necessary for their subsequent releases.

### 3.3. 3D Porous Scaffold Generated by Freeze-Casting

All of the above findings indicate the possibility, via a 3-fluid nozzle spray dryer approach, to prepare “*à la carte*” core-shell particles based on substituted biomimetic apatites, exhibiting the desired substituting ions both in the inner core and the outer shell. Such particles may then be seen as particularly well suited for the setup of bone regeneration biomaterials with smart releasing properties. In order to illustrate this fact, we aimed at preparing 3D composite scaffolds based on a polymeric matrix with embedded core-shell particles.

**Figure 7:**
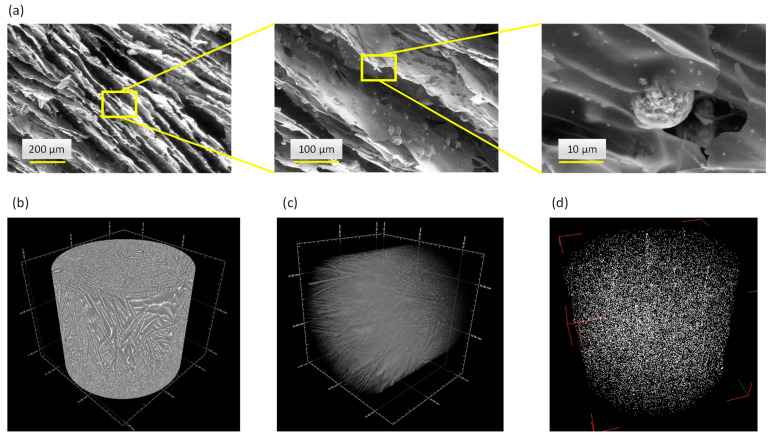
Freeze-cast 3D porous scaffolds associating alginate and core-shell particles: SEM micrographs (**a**) and µCT image (**b**) of the alginate scaffold with embedded [(Cu^2+^)Ag^+^] particles, (**c**) µCT ROI reconstruction of the porous network and (**d**) spatial distribution of the [(Zn^2+^)Cu^2+^] core-shell particles embedded in the alginate matrix.

In this work, sodium alginate was selected as polymer matrix due to its biocompatible and bioresorbable properties. Considering the metastable character of biomimetic apatite nanocrystals, the freeze-casting methodology was chosen to avoid thermal alteration for porosity template removal, while providing additional advantageous characteristics such as an oriented open porosity, close to natural cortical bone structure and favorable to cell colonization and neo-angiogenesis. The selected SEM micrographs of the scaffolds generated with a mass ratio of 40:60 between the core-shell apatite particles and alginate are reported in Figure 7a, along with µCT observations (Figure 7b). These observations confirm that an oriented open porosity was indeed obtained, typically along the height of the cylindrical scaffolds. This may be explained by the propagation of the freezing front of the ice from bottom to top during the freeze-casting process, as reported previously [57,58,59,60,61,62]. In particular, Figure 7c illustrates the porous network as seen by µCT via region-of-interest (ROI) image reconstructions to enhance the porous architecture within the materials: these observations evidence an array of mostly parallel polymer walls. Both the SEM and µCT show embedded particles retaining their initial spherical morphology. In a complementary way, µCT ROI reconstructions allowed us to also examine the 3D spatial distribution of the core-shell apatite particles within the alginate scaffold (Figure 7d), confirming their homogeneous dispersion across the three dimensions of space. The micronic size of the particles associated with their potential alginate coating (ionic interaction) prevent their aggregation and their further sedimentation.

### 3.4. In Vitro Assays

One system particularly appealing in this work appears to be the [(Cu^2+^)Ag^+^] core-shell biomimetic apatite particles, taking into account the high antimicrobial properties of Ag^+^ ions and the polyvalent osteogenic/anti-inflammatory/pro-angiogenic character of Cu^2+^ ions. The selected in vitro tests were thus run here, in a preliminary approach, with two main goals: first, to verify the non-toxic character of Cu- and Ag-substituting by demonstrating the biocompatibility of Cu- or Ag-substituted biomimetic apatites; and second, to validate the antibacterial properties offered by the Ag-substituting, since Ag^+^ ions will be the first ions to be released from the engineered particles formulated in this study.

In the first stage, the cytocompatibility of Cu- and Ag-substituted apatite compounds (0.2 and 0.5 % initial substitution rates, see Table 1) in powder form was tested on pre-osteoblastic MG63 cells. This cell line presents phenotypic stability and shows some matching behavior with normal cells, namely a variety of common osteoblastic markers and sensitivity to hormonal response, being suited to be used as an in vitro model for testing biomaterials for bone applications [63,64].

**Figure 8:**
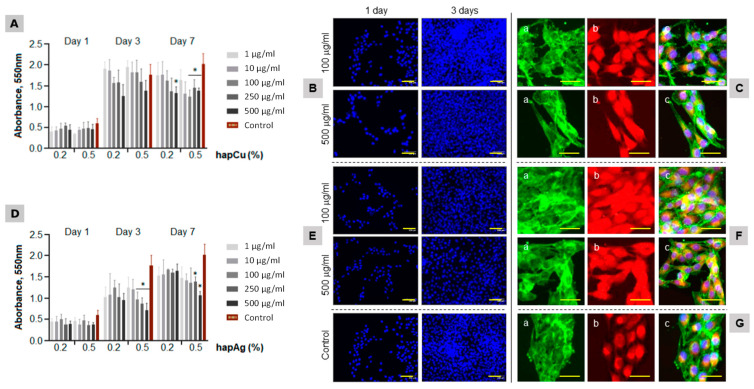
Behavior of MG63 osteoblastic cells exposed to hapCu (**A**–**C**) and hapAg (**D**–**F**) powders (1 to 500 μg/mL). Control ((**G**); standard tissue culture plate). Cell viability/proliferation ((**A**,**D**); days 1, 3 and 7) and cultures stained for the nuclei ((**B**,**E**,**G**); blue, days 1 and 3), F-actin cytoskeleton staining ((**C**(**a**),**F**(**a**),**G**(**a**)), green, day 3), mitochondria tracking ((**C**(**b**),**F**(**b**),**G**(**b**)); red, day 3) and triple staining ((**C**(**c**),**F**(**c**),**G**(**c**)); day 3). * Significantly different from control, *p* ≤ 0.05. Bar = 50 μm.

The adherent MG63 cells were exposed to the substituted apatite powders at increasing concentrations (from 1 to 500 µg/mL) for periods up to 7 days. MTT assay was used to measure the cell metabolic activity, also providing information on cell proliferation due to the linear relationship between cell activity and absorbance. The cells were also characterized for morphology, F-actin cytoskeleton and mitochondria tracking. The results are summarized in Figure 8.

The control cultures (Figure 8G) showed an increase in the number of viable cells (both taking into account direct viability and proliferation) from day 1 to day 3, with a tendency for stabilization afterwards. The cells exposed to hapCu powder had a similar pattern. At day 1, the values were similar to the control. For longer exposures, a trend for a concentration-dependent decrease was noticed, although significant differences compared to the control were seen only for 7-day exposure to 500 µg/mL (Figure 8A). The low toxicity was confirmed by representative fluorescence microscopy images of the cultures exposed to hapCu 0.5% at 100 and 500 µg/mL (Figure 8B,C). The images illustrate the high proliferation rate (Figure 8B, cells stained for the nucleus), the uniform distribution of the F-actin cytoskeleton with increased staining at the cell edges (Figure 8C(a)), and the great abundance of mitochondria (Figure 8C(b)), i.e., the organelles that generate most of the cell’s supply of ATP, which is used as a source of chemical energy. The triple cell staining (Figure 8C(c)) showed well-organized cultures of interacting healthy cells, with elongated morphology, a prominent nucleus and a perinuclear localization of the mitochondria. The results confirm the negligible toxicity of hapCu, which is in line with a variety of previous studies addressing the biological performance of Cu-incorporated biomaterials [17,21,22,65], which further demonstrates an improvement of the angiogenic and osteogenic properties of the substituted materials [23,24,25,26,27,28,66,67].

The MG63 cells exposed to hapAg powders presented a gradual increase in the proliferation over the culture period (Figure 8D). A concentration-dependent decrease was noticed, particularly after 3 days of exposure to the highest tested concentrations. However, it is noteworthy that the cells were able to recover during the exposure and, at day 7, significantly decreased values were observed only in cultures treated with 250 and 500 µg/mL hapAg 0.5%. The fluorescence images also illustrated the high proliferation rate (Figure 8E), the organization of the F-actin cytoskeleton (Figure 8F(a)), and mitochondria tracking (Figure 8F(b)). Despite the lower MTT reduction values observed in the cultures exposed to 500 µg/mL hapAg 0.5%, the triple-staining of the cultures showed a healthy morphology, cell-to-cell interaction and an organized culture (Figure 8F(c)).

**Figure 9:**
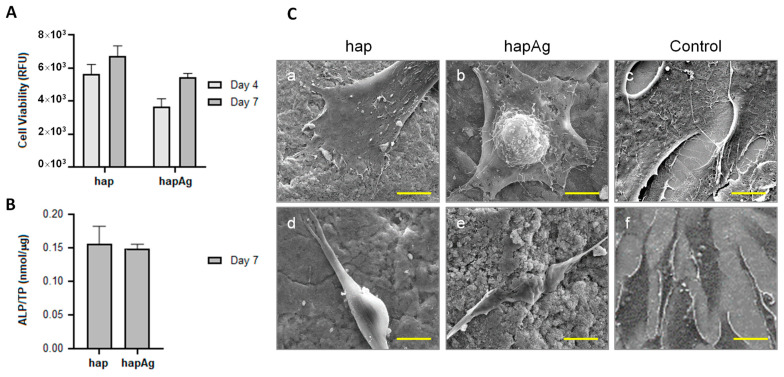
Behavior of MG63 osteoblastic cells cultured over hapAg pellets. Viable cell count ((**A**); days 4 and 7), alkaline phosphatase activity ((**B**); day 7) and SEM observation ((**C**); day 4). Control (standard tissue culture plate). Bar = 5 μm (**a**–**c**), 10 μm (**d**), 2 μm (**e**) and 20 μm (**f**).

Additional tests were carried out on pelletized Ag-substituted apatite samples to increase the concentration of apatitic particles/Ag^+^ ions in contact with the cells and further approach real in vivo conditions. Indeed, while Cu^2+^ ions are naturally present in vivo (e.g., associated to metalloproteins), Ag^+^ ions are not naturally present in the body and deserve further attention. The MG63 osteoblastic cells were cultured over hapAg 0.5% for a period of 7 days, and the cell response was compared to that observed in the hap pellets (Figure 9). The number of viable cells increased throughout the culture time over both materials. Slightly lower values were measured on the hapAg pellets, especially at day 4, but it should be emphasized that the cell growth rate from day 4 to day 7 was higher on the Ag-substituted samples, suggesting a progressive cell recovery approaching the viable cell count values reached for hap (Figure 9A). An important indication of the low toxicity of pelletized hapAg is that ALP activity was similar to that on the hap samples (Figure 9B). ALP is an abundant membrane-bound glycoprotein in osteoblasts and is considered an early marker of osteoblast differentiation. This enzyme hydrolyzes inorganic pyrophosphate (a calcification inhibitor), having an essential role in the initiation of the bone matrix mineralization [68]. SEM representative images of the colonized pellets (Figure 9C) showed well-spread cells closely interacting with the underlying material topography in both material samples. These results strongly suggest that the Ag^+^ apatite substituting conditions in hapAg 0.5% did not result in cytotoxicity.

In the second stage, the potential antibacterial properties of the Ag-substituted biomimetic apatite pellets were examined by selecting two major bacterial strains relevant to bone infections, namely *E. coli* (Gram negative) and *S. aureus* (Gram positive). In these experiments, an appropriate bacterial suspension was poured over the hap and hapAg pellets and the antibacterial activity was assessed against the bacteria in suspension (planktonic; rezasurin assay) and those adhered to the material surface (sessile; rezasurin assay and CFUs counts), after an incubation period of 24 h.

**Figure 10:**
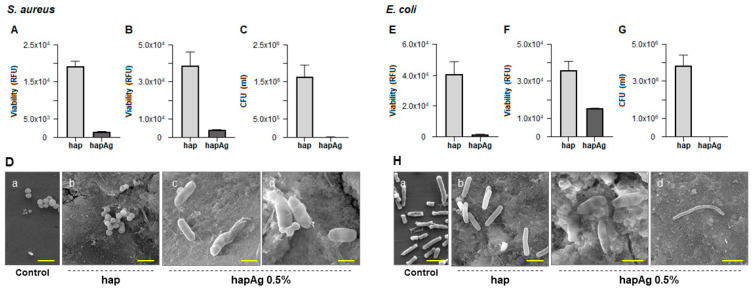
Antibacterial activity of hapAg pellets against *S. aureus* (**A**–**D**) and *E. coli* (**E**–**H**). Viability of planktonic bacteria (**A**,**E**), sessile bacteria (**B**,**F**), CFUs counts of sessile bacteria (**C**,**G**) and SEM observation (**D**,**H**). Control (standard tissue culture plate). Bar: (**D**)—2 μm (**a**,**b**), 1 μm (**c**) and 0.5 μm (**d**); (**H**)—2 μm (**a**,**b**), 1 μm (**c**) and 10 μm (**d**).

Compared to the hap pellets, more than 90% inhibition on the viability of *S. aureus* was observed both on planktonic (Figure 10A) and sessile (Figure 10B) bacteria over hapAg. Due to the relevance of the adhered bacteria in the formation of biofilms, these were also evaluated by CFUs counting, and the results show a negligible ability in the formation of colonies from the adhered bacteria collected from the Ag-substituted apatite (Figure 10C). The antibacterial activity was confirmed by SEM observation of the infected materials. *S. aureus* growing in the hap pellets were able to form regular colonies of cells displaying the typical round morphology and size (about 0.5 to 1 µm in diameter, Figure 10D(a)). By contrast, the few bacteria present on the hapAg pellets exhibited an atypical elongated and altered morphology (Figure 10D(b)) and, in addition, the formation of bacterial chains (Figure 10D(c)), suggesting an inability to further proceed to cell division, a process that prevents the formation of a biofilm. The difference observed in the inhibition results shown in Figure 10D(b,c), both addressing sessile bacteria, is due to the different degree of accuracy of the two methods. Resazurin assay accesses the metabolic activity of viable cells by fluorescence evaluation, providing reliable results of the bacterial viability [69]. However, CFUs counts, accounting for the bacteria that are able to form a colony, would prove both the cell viability and functionality, and is therefore the most accurate method [66].

Ag-substituted apatites were also endowed with a significant antibacterial activity against *E. coli*. Compared to hap, the viability of planktonic bacteria was negligible (Figure 10E), and a significant reduction was seen in the adhered bacteria (Figure 10F). CFUs counts of these showed a total inhibition in the formation of colonies (Figure 10G). The difference between the results presented in the two last figures addressing sessile bacteria results from the reasons mentioned above. The SEM images (Figure 10H) showed clear differences in the morphology and pattern of growth of the bacteria adhered to hap and hapAg. On hap, cells with typical rod-shaped morphology and size (about 2 µm long and 0.25 to 1 µm width, Figure 10H(b)) were observed, contrasting with those present on hapAg displaying greatly altered morphology (Figure 10H(b)) and the formation of long bacteria chains unable to proceed to cell division (Figure 10H(c)).

Ag^+^ ions are a versatile antibacterial agent showing low tendency toward the development of bacterial resistance, and are particularly effective against polymicrobial colonization associated with biomaterial infection. The associated mechanisms appear to result from the damage of the cell wall and cell membrane (due to electrostatic attraction with these negatively-charged structures) [70,71,72,73], binding with membrane proteins involved in the trans-membrane energy regeneration and ion transport, interaction with the phosphate groups of DNA causing bacterial degeneration and the inability to replicate [71,73,74,75,76] and the intracellular formation of reactive oxygen species (ROS) ultimately leading to cell death [67,70,71,74,77]. Furthermore, several in vitro studies performed in Ag-substituted apatites reported that Ag^+^ ions play an important role in preventing initial bacterial adhesion [78,79,80], supporting the results found in the present work regarding the significant antibacterial effect in sessile bacteria. Therefore, the selection of antibacterial ions such as Ag^+^ in the composition of the outer shell of the engineered core-shell particles envisioned in the present work appears particularly suitable.

## 4. Conclusions

The objective of this work was to propose a versatile bone-inspired system capable of providing both spatial and temporal control of multiple bioactive ions release. To this aim, we report here for the first time the production of multifunctional core-shell particles made of biomimetic apatite, with an external layer incorporating an antibacterial ion, such as Ag^+^ or Cu^2+^, and an internal core rich in pro-angiogenic or osteogenic ions, such as Cu^2+^ or Zn^2+^. These particles were obtained by an innovative 3-fluid nozzle spray drying approach, and the core-shell substructure was evidenced by combined FEG-SEM/FIB/EDS analyses. The underlying concept of such core-shell micron-sized particles is thus to expose a first substituting ion in the outer shell (typically an antibacterial ionic species) and a different substituting ion in the inner core of the particles (e.g., angiogenic), as this spatial control of ions localization is needed in in order to control the sequence of events to occur in vivo after implantation. The effective antibacterial properties and non-cytotoxic character of substituted biomimetic apatites were confirmed in vitro. Such particles were then distributed in a homogeneous way, as demonstrated by µCT, within a biopolymer 3D scaffold made of alginate, exhibiting an oriented open porosity favorable for cell colonization/bone healing. The partial embedding of these core-shell structures in the walls of the polymer scaffold will provide some additional mechanical stability to the particles. From a chemical viewpoint, the latter are expected to progressively dissolve upon cell activity after implantation, and dependently of the local microenvironment.

Such 3D pieces could prove helpful for bone regeneration purposes with the ability to anticipate beforehand the behavior of the implanted biomaterial, for example with a first antimicrobial action for eradicating pathogenic microorganisms followed by a second ion release to favor bone repair and cell activity. Now that this proof of concept to obtain dually-substituted bio-inspired apatite core-shell structures has been established, the future work prior to further biological evaluations will be dedicated to a systematic study of the ions release profiles, which are bound to be modulated by the local conditions (medium, inflammatory acidic pH, dynamic/static, etc.) and requires a specific study.

## Figures and Tables

**Table 1:** Listing of biomimetic apatite samples and substituting rates studied in this work, with theoretical and experimental substituting ratios.

Sample *	% Substituting Ratio (Theoretical)	% Substituting Ratio ** (Experimental)
hapAg 0.2%	0.2	0.2
hapAg 0.5%	0.5	0.4
hapAg 5%	5	2.5
hapCu 0.2%	0.2	0.2
hapCu 0.5%	0.5	0.5
hapCu 10%	10	9.4
hapZn 10%	10	10.5

* The indicated percentage relates to the initial molar content of the substituting ion in the precipitating medium. ** Experimental data are within 1% relative error.

## Data Availability

Data are available by contacting the corresponding author(s).

## Supplementary Materials

The following supporting information can be downloaded at: https://www.mdpi.com/article/10.3390/nano13030519/s1: Figure S1: SEM observations of selected precipitated biomimetic apatite samples for reference (undoped) and single-ion doping. Figure S2: (a) XRD and (b) FTIR analyses on core-shell particles. Figure S3: Particle size distribution spanning in the range 1.5–15 µm with a (Gaussian) statistical mode, with a mean diameter to 6.0 ± 3.3 µm. Figure S4: Castaing’s formula.
