# Supplementary material for: Toward Smart Biomimetic Apatite-Based Bone Scaffolds with Spatially Controlled Ion Substitutions
Source: Nanomaterials (Basel). 2023 Jan 28;13(3):519. doi: 10.3390/nano13030519 (PMC9919144; doi:10.3390/nano13030519)
Supplement: Supplementary file 1 [file nanomaterials-13-00519-s001.zip › nanomaterials-2181458-supplementary.pdf]

Supporting Information

Edoardo Cianflone [1,2], Fabien Brouillet [2], David Grossin [1], Jérémy Soulié [1], Claudie Josse [3], Sanjana Vig [4,5], Maria Helena Fernandes [4,5], Christophe Tenailleau [2], Benjamin Duployer [2], Carole Thouron [1] and Christophe Drouet [1,*]

[1] CIRIMAT, Université de Toulouse, CNRS, INP-ENSIACET, 31030 Toulouse, France
[2] CIRIMAT, Université de Toulouse, CNRS, UT3 Paul Sabatier, 31062 Toulouse, France
[3] Centre de Microcaractérisation Raimond Castaing, Université de Toulouse, UPS, CNRS, INP, INSA, 31400 Toulouse, France
[4] Faculdade de Medicina Dentaria, Universidade do Porto, Rua Dr Manuel Pereira da Silva, 4200-393 Porto, Portugal
[5] LAQV/REQUIMTE, University of Porto, 4160-007 Porto, Portugal
[*] Correspondence: christophe.drouet@cirimat.fr

non-substituted biomimetic apatite (hap-6d, ref)

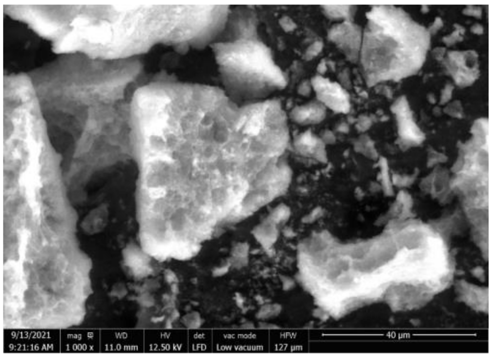

hap-1d 10%Cu

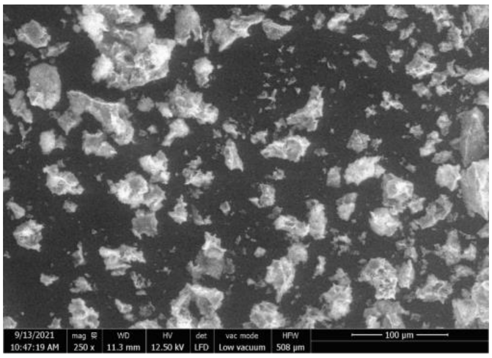

hap-1d 15%Zn

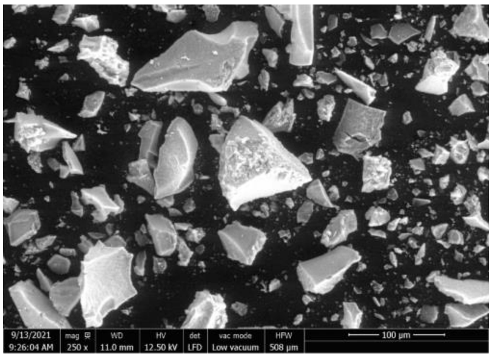

Figure S1: SEM observations of selected precipitated biomimetic apatite samples for reference (undoped) and single-ion doping.

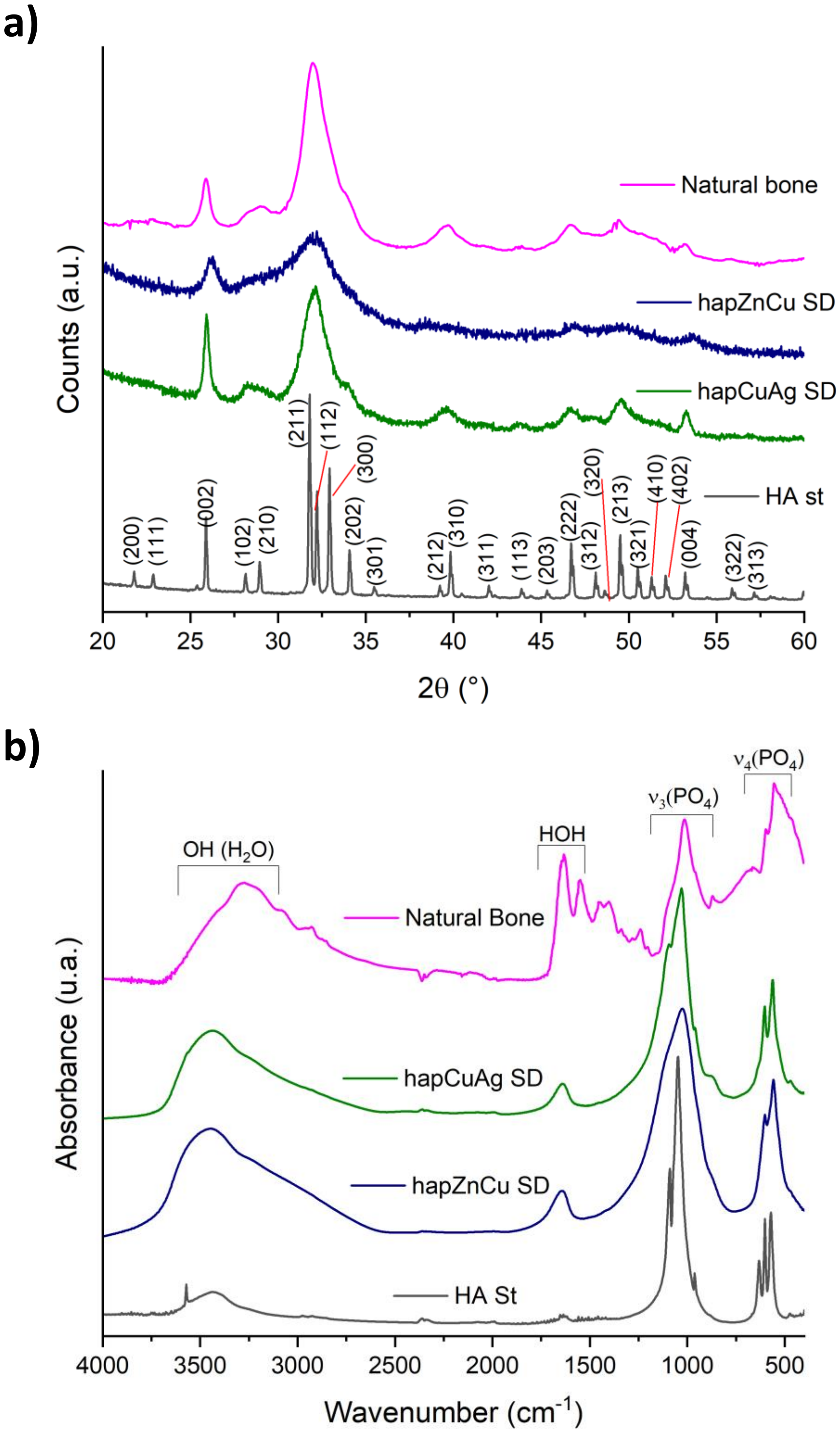

Figure S2: (**a**) XRD and (**b**) FTIR analyses on core-shell particles.

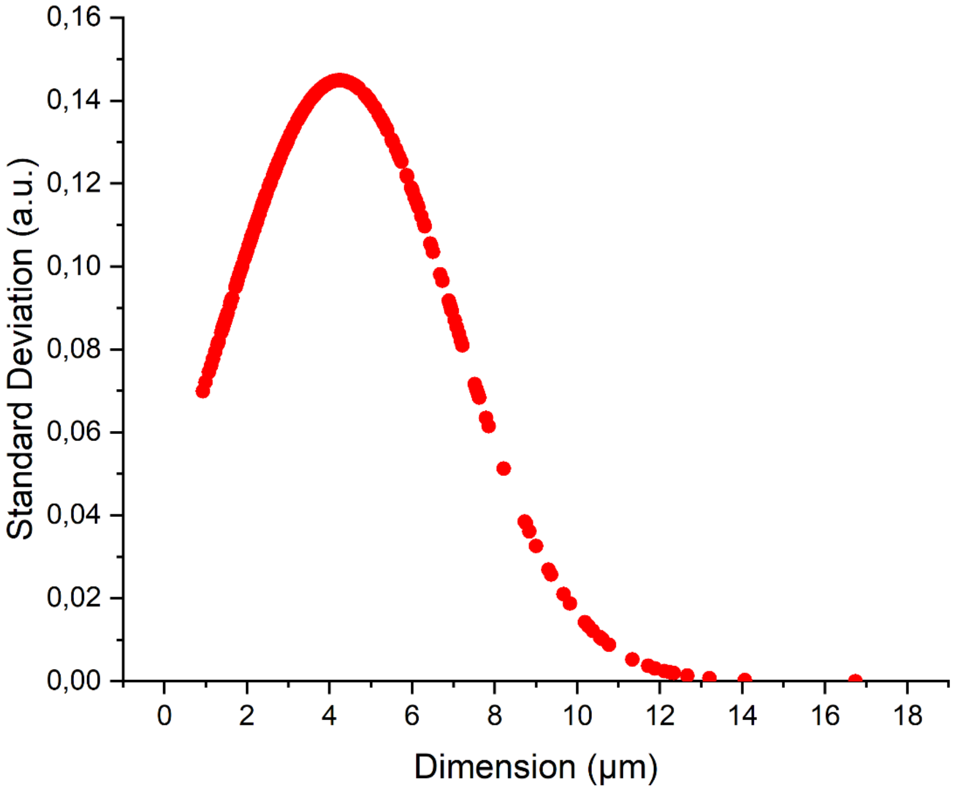

Figure S3: Particle size distribution spanning in the range 1.5-15 µm with a (Gaussian) statistical mode, with a mean diameter to 6.0 ± 3.3 µm.

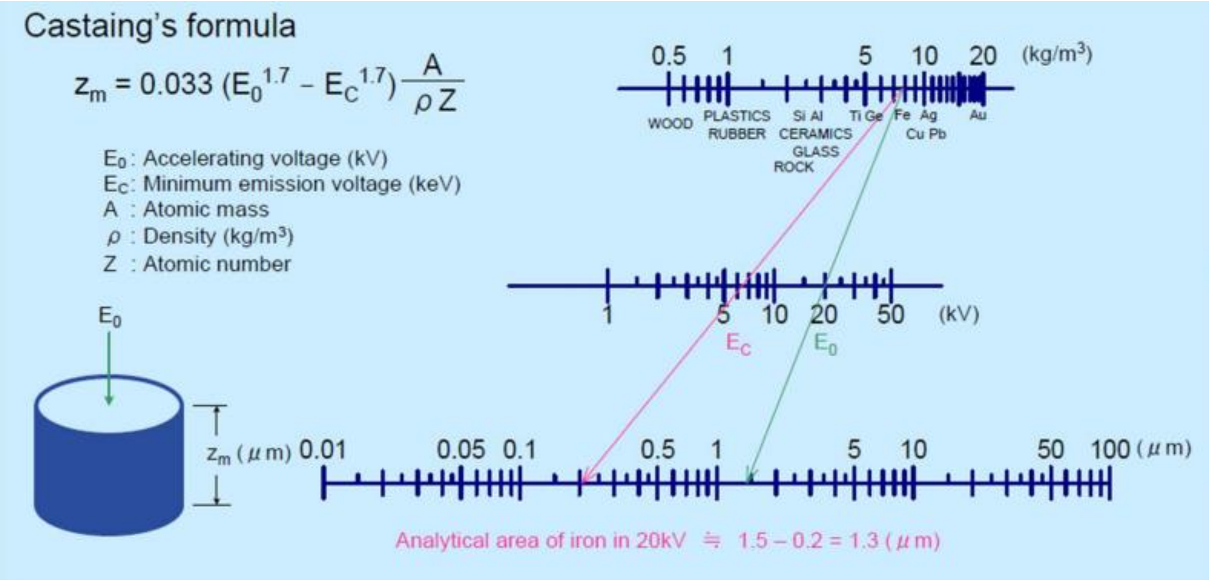

Figure S4: Castaing's formula.
